# New Works on Botany

**Published:** 1842-05

**Authors:** 


### NEW WORKS ON BOTANY.

We learn from a late number of the New York Medical Gazette, that the second part of the *Flora of North America*, by Drs. Torrey and Gray, was published on the first of April. The same Journal states that a new *Botanical Text-Book*, from the pen of Dr. Gray, will appear early in the present month. It will treat briefly of the structure and physiology of plants, the natural families of the vegetable kingdom, and contain a list of the principal plants used in medicine and the arts. The author's enviable reputation as a man of science, is a guarantee for the excellency of the forth-coming work. The Gazette says:

"This work will be of great value to those medical students who desire to gain a knowledge of botany, and we rejoice to say that the number of such is daily increasing. Indeed, we believe that the chief reason why botany has been so much neglected by the profession in our country, is, that the means of studying it scientifically have not been afforded them; the only text books within reach of the student, being of a character to disgust him with a branch of natural science, which, if properly presented to his view, could not but prove as eminently attractive as it is practically useful. This great difficulty will now be removed. Dr. Gray is well known, both in this country and abroad, as a man of true science, and we are rejoiced that a gentleman who has shown, by previous publications, that he is capable of *higher things*, has consented to prepare a text book of his favorite science. We bespeak for his work the favor of his profession. We have no doubt that it will be generally used in our medical institutions. We would suggest to those of our brethren who have a voice in the control of schools, academies, and other literary institutions, that they will do science a service by introducing Dr. Gray's Text Book."

